# A Space-Variant Visual Pathway Model for Data Efficient Deep Learning

**DOI:** 10.3389/fncel.2019.00036

**Published:** 2019-03-26

**Authors:** Piotr Ozimek, Nina Hristozova, Lorinc Balog, Jan Paul Siebert

**Affiliations:** Computer Vision for Autonomous Systems Group, School of Computing Science, University of Glasgow, Glasgow, United Kingdom

**Keywords:** data efficiency, deep learning, retina, foveated vision, biological vision, egocentric perception, robot vision, visual cortex

## Abstract

We present an investigation into adopting a model of the retino-cortical mapping, found in biological visual systems, to improve the efficiency of image analysis using Deep Convolutional Neural Nets (DCNNs) in the context of robot vision and egocentric perception systems. This work has now enabled DCNNs to process input images approaching *one million pixels* in size, *in real time*, using only consumer grade graphics processor (GPU) hardware *in a single pass of the DCNN*.

## 1. Introduction

Deep Learning methods have revolutionised signal and image analysis, and indeed end-to-end approaches to training these networks can achieve the state-of-the-art in vision-based control (Viereck et al., 2017; Morrison et al., 2018) and recognition for robotics. However, a real obstacle to the practical adoption of DCNNs is their requirement for very large training data sets and their inability to scale to process image matrices of greater than approximately 300 × 300 px in a single pass. We address this issue directly by adopting a computational model of the space-variant, i.e., *foveated*, visual processing architecture found in the mammalian vision system (Schwartz, 1977). Our 50K node retina-preprocessor enables current DCNN networks to process input images of 930 × 930px in size, using only consumer grade graphics processor (GPU) hardware, *in a single pass of the DCNN* and this retina pre-processing approach has the potential to scale to accommodate larger input image sizes. In addition, this pre-processor mapping confers a degree of scale and rotation invariance to the transformed images facilitating a number of perception tasks, reducing the parameter size and computation required to train a DCNN.

The above visual processing limitations of current DCNN implementations appear to have been addressed effectively in biological vision systems. The visual pathway from the retina to V1 itself implements space variant sampling in the retina to afford a very substantial data reduction and also a key spatial transformation, the retino-cortical mapping (Schwartz, 1980). This transformation affords a number of additional signal simplifications, including a degree of scale and rotation invariance.

In this paper we present a retina-DCNN pipeline that confirms our hypothesis that DCNNs are capable of learning in *cortical-space* and undertaking inferences in a single pass of this space. We detail the results of experiments that confirm it is possible to harness simple functional computational models of the space-variant retino-cortical mapping to improve the efficiency of DCNNs and demonstrate combining our latest functional retina models with this mapping. Accordingly, by applying a model of the retino-cortical transform, as a pre-processing step, to produce a much smaller *cortical image*, current DCNNS are capable of processing, in a single pass, cortical images generated from retina input images of the order of one million pixels. The above efficiency improvement, and those predicted for the other known processing transformations in the visual pathway, appear to have the potential to solve many of the data efficiency issues in adopting DCNNs in practice.

Within the retina itself, opponent ganglion cells compute a colour space potentially capable of simplifying tasks such as texture perception and contour detection. Motivated by this signal simplification property, we present a GPU accelerated high resolution software retina implementation that incorporates basic models of opponent colour and intensity P-pathway ganglion cells.

In addition to the above contributions, we outline a number of practical examples of the utility of the retina-DCNN combination: an egocentric perception system based on human eye-tracking to provide retina gaze control for training and object recognition, pan-tilt control of a robot wrist-mounted camera, retina enabled camera interfaces and retina data management tools.

We now overview the relevant properties of early mammalian visual pathway and the retino-cortical mapping that are relevant to supporting efficient visual computations, in the next section.

## 2. Prior Research in Retino-Cortical Mapping Models of the Visual Pathway

### 2.1. The Mammalian Vision System

Any perceived light entering the eye-ball stimulates a hemispherical layer of photoreceptor cells. These cells are densely packed in the central *foveal* region of the retina and are more sparsely distributed in its peripheries (Curcio et al., 1990). Of the two types of photoreceptor cells, rods & cones, we have only considered the cones which facilitate *photopic* colour vision and discern fine detail. Since the rods are not present in the fovea, we have not investigated the rod pathway within the scope of this work which focuses on robot vision systems.

The signals produced by the photoreceptor cells are sequentially pre-processed through up to four different neuron types before exiting the retina and reaching the brain. It is important to note that the typologies of these intermediate retinal neurons coarsely follow the foveated topology of photoreceptor cells, i.e., are held in retinotopic registration, and that it is these topologies combined with visual attention mechanisms that enable the retina to control the rate of visual information passed onto the brain, Curcio and Allen (1990). It has been estimated (Schwartz, 1993), that if our eyes sampled our whole field of view at the foveal resolution, our visual cortex would have to be larger by several orders of magnitude to accommodate this computational load.

An important feature of the retina's neural architecture is the concept of *receptive fields*. The final intermediate retinal neurons that relay the visual signal to the brain are called retinal ganglion cells (RGCs), and most of them receive information from multiple neighboring photoreceptor cells through intra-retinal pathways. Those local clusters of photoreceptor cells comprise the RGC's receptive field. The sizes of these receptive fields increase with the distance (*eccentricity*) from the fovea, with the foveal RGCs only relaying information from individual photoreceptor cells (Hubel et al., 1995). Different RGCs have receptive fields of different response profiles depending on their specific function, which can range from discerning detail to computing the magnitude of differential motion (Ölveczky et al., 2003, 2007). Our basic retino-cortical mapping work is presented in the next section and more detailed RGC models are also presented in section 6.1.

### 2.2. The Retino-Cortical Transform

**Figure 1:**
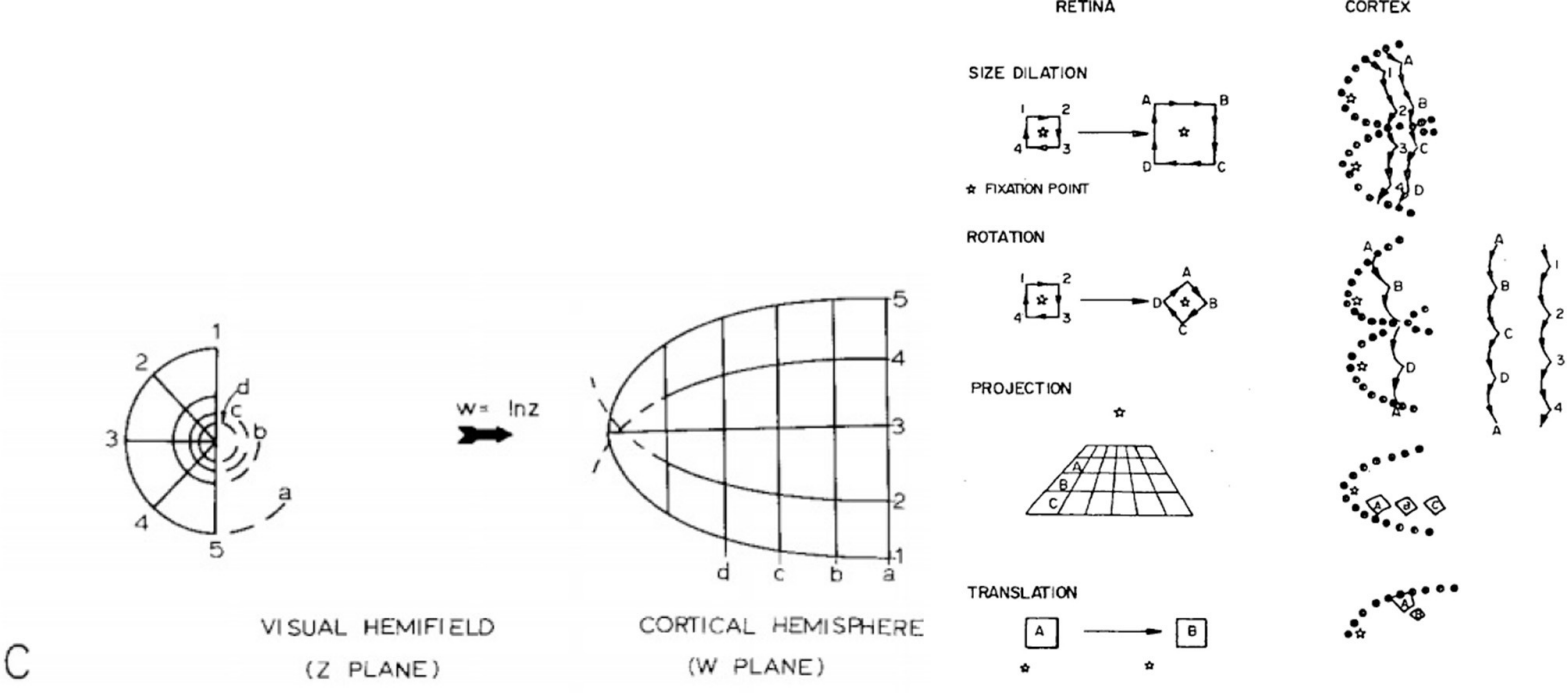
**Left:** Global retinotopic mapping (Schwartz, 1977); **Right**: Scale rotation and projection invariance for a log(z+1) cortical mapping, taken from Schwartz (1980).

The RGCs pass the visual signal from the retina to the primary visual cortex (*V1*). The signal from each eyeball is split into two halves and each half is projected separately via a structure known as the Lateral Geniculate Nucleus (LGN) onto each hemisphere in V1, where it is can be observed as a form of spatial complex logarithmic mapping, similar to that in Figure 1, Left. This mapping appears to be the format in which our brains process vision and it could potentially be one of the mechanisms that facilitate scale invariance in biological vision systems (Schwartz, 1977).

The key phenomenon that Schwartz models in Figure 1, Left above is *cortical magnification*. The densely sampled fovea achieves the effect of appearing to be magnified in the cortex when compared the retina periphery, which appears to be progressively compressed as a function of retinal eccentricity. This of course reflects the photoreceptor sampling density within the retina beyond the fovea, which decreases exponentially.

The above also gives rise to the data-reduction property of the retino-cortical mapping and hence its resultant data efficiency. In *O* notation used to specify computational complexity, i.e., *order of* , a uniform areal sampling of the retina would require *O(r*^2^*)* receptive fields while a log-polar mapping reduces this to *O(r)*, (Wilson and Hodgson, 1992). Indeed if we consider the number of receptive fields required to sample a *contour* this becomes *constant* (Wilson, 1983, 1985). As a consequence, the space-variant structure of the retina provides foveal full resolution and the ability for a human to “thread a needle”, while simultaneously monitoring ˜180° (combined binocular vision) of the environment. In effect nature has equipped us with a static “zoom lens”.

Schwartz (1980), proposed that the retinal samples are mapped to the cortex via a form of log-polar mapping. A number of further perceptual gains arise from the above architecture: a *pure* log-polar mapping, Equations 1, 2, results in a local edge contour segment translating along the θ axis under input rotation about a fixation point, while scale change centred on the fixation point causes a local contour segment to translate along the ρ cortical axis. In addition, peripheral objects located on a common ground plane (with respect to an observer) also retain their local shape appearance, i.e., exhibit projective invariance in the cortex. These three invariance properties are illustrated in Figure 1, Right (for a log (z+1) cortical mapping) and are referred to as *edge invariance*, since the *conformal* log-polar mapping only preserves local angles, and the local shape of the contour, but not its global shape. However, this architectural organisation is clearly of significance in terms of scale, rotation and projective invariance for feature descriptors operating over the whole cortical field (Wilson and Hodgson, 1992), and likewise offers potential utility for the interpretation of stereopsis and texture perception in the striate cortex (Schwartz, 1981).

Unfortunately, a pure log-polar transform gives rise to a non-uniform fovea and a sampling density singularity at the centre of the fovea. In order to avert this topological crisis, Schwartz proposed that a small constant, α, be introduced to the horizontal mapping axis, Equations 4–7. When α is small compared to the horizontal Field of View (FoV), the fovea then becomes approximately linear (i.e., shift invariant) in sampling and the periphery approximates a log-polar mapping. This mechanism also gives rise naturally to the split in the retinal vertical meridian observed in the mammalian eye-brain mapping, as described in Equations (6, 7), and illustrated in **Figure 5**. While the mapping no longer produces a purely orthogonal output map in response to scale input transformations, the effect of such input changes is to produce a smoothly continuous warp of input contours along streamlines in cortical space. Accordingly, the continuity of these transformations still reduces the size of the cortical pattern space that any subsequent perceptual system must learn to accommodate invariance to input scale and rotation changes.

Further implications can be found for interpreting egocentric optical flow fields, where time-to-impact can be read directly from the ρ axis on the cortex and deviation from a horizontal flow field can be interpreted as being due to the presence of non-stationary objects in the FoV (Rojer and Schwartz, 1990).

Schwartz (1980) also demonstrates how his complex-log mapping can be used to explain the cortical magnifications observed in a number of mammalian species. Johnson (1986, 1989) extends Schwartz's analysis by demonstrating that the 3D nature of retina and cortex that should be taken into account in order to explain fully the mapping and confirms this hypothesis with biological data. However, Johnson's extended mapping is beyond the scope of this paper.

The foveated nature of the mammalian visual architecture also impacts visual search, affording an “attentional spotlight” that suppresses extraneous details while retaining pertinent diagnostic image features, e.g., one recognises an individual from the gross structural features of their face, as opposed the detailed arrangement of their skin pores or strands of their hair. Hence, full visual acuity is directed by the visual system to interrogate the scene where required, while gross structural features provide both context and the necessary diagnostic information for visual interpretation of the scene, and specific items, or regions, within it. In an earlier implementation of the retina described here based on the use of SIFT (Scale Invariant Feature Transform) (Lowe, 2004), visual descriptors for image matching, we found that the retina actually improved recognition rates over the use of SIFT processing at full-resolution. We attribute this result to the retina smoothing out irrelevant detail, when it is directed to classify diagnostic locations (Ram and Siebert, 2011).

### 2.3. Functional Retina Models

Commencing with the pioneering work by Schwartz and his contemporaries, many implementations of vision systems have been reported which adopt log-polar mappings (for example: Weiman, 1988; Bolduc and Levine, 1997; Gomes, 2002; Balasuriya and Siebert, 2006). A relatively recent example of a space-variant vision system was proposed by Pamplona and Bernardino (2009) who devised a method of generating foveated images using overlapping Gaussian receptive fields and a technique for performing conventional image processing functions on such images using matrix operations. This work makes a strong case for using Gaussian receptive fields rather than superpixels as an accurate and computationally efficient way of performing retinal sampling, although the resultant images produced by their method suffer from a number of artefacts.

We have based our work on an earlier retina model developed by Balasuriya (2006) which also employs Gaussian receptive fields. Balasuriya reports a complete vision system based on a self-organised software retina that combines mechanisms for retina tessellation generation, retina sampling, feature extraction and gaze control, Balasuriya and Siebert (2003, 2006). The output from this retina was originally processed using SIFT-like descriptors and directed by means of a SIFT keypoint based gaze control system and is now processed using DCNNs in the work reported here.

#### 2.3.1. Balasuriya's Retina

**Figure 2:**
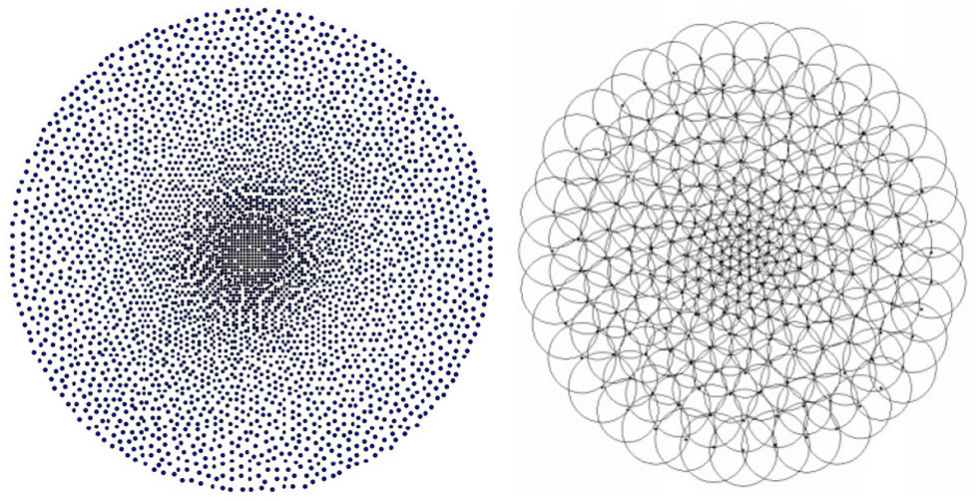
**Left:** The 4196 node tessellation used in this paper. **Right:** Gaussian receptive fields on top of a retina tessellation, taken from Balasuriya (2006).

A central issue is how to generate a retina tessellation in such a way that no local discontinuities, distortions or other artefacts are produced when making a transition between the retina's quasi-uniform fovea and its log-polar periphery. To solve this problem Balasuriya employs a self-similar neural network (Clippingdale and Wilson, 1996). This method relies on a network of N units jointly undergoing random translations to produce a tessellation with a near-uniform dense foveal region that seamlessly transitions into a sparse periphery, Figure 2, Left. Each node in the resultant tessellation defines the location of a receptive field's centre. The receptive fields somewhat follow the biological retina's architecture; they all have a Gaussian response profile the standard deviation of which scales linearly as a function of local node density, which in turn scales statistically with eccentricity, Figure 2, Right, due to the stochastic nature of the tessellation. This scaling balances between introducing aliasing at the sparsely sampled peripheries and super-Nyquist sampling at the densely sampled foveal region. Since the receptor scaling varies locally with node density it is possible to have receptive fields at the same eccentricity with slightly different field diameters, as found in nature, to avoid “holes” in the receptor layout.

The values sampled by the receptive fields are stored in an *imagevector*, which is essentially a one-dimensional array of intensity values. After defining the retina in Balasuriya (2006), a scale-space retina pyramid is presented which is used to extract corner-based saliency information that drives the gaze control mechanism. The saliency information is extracted from the receptive fields, stored in imagevector format, and then back-projected onto the saliency map which is then normalised based on the Gaussian field intensity. An inhibition-of-return map employing a similar mechanism has been adopted to prevent the retina from continuously fixating upon the same location. Having computed the saliency map, the retina saccades to the location with the highest value of the difference between the saliency map and the inhibition-of-return map and the whole process is repeated for each retina fixation that follows. The work presented here is based on an improved version of Balasuriya's retina implementation and conceptual aspects of his gaze control mechanism.

### 2.4. A 4196 Node Retina Implementation

Our first attempt at coupling the software retina described in section 2.3.1 to a DCNN utilised a tessellation comprising *N* = 4, 196 nodes, for *r*_*fov*_ = 0.1, and required *N*_*iter*_ = 20, 000 iterations to stabilise, Figure 2, Left and is defined by the following tessellation parameters:

- *N*- number of nodes in the retina tessellation
- *r*_*fov*_ - the fovea's radius as a fraction of the tessellation's radius.
- *N*_*iter*_ - number of iterations for self-organisation of the self-similar neural network, used in generating the retina tessellation.

Receptive field parameters:

- *dist*_5_ - the mean pixel distance of the 5 central foveal nodes to their 5 closest neighbors
- σ_*base*_ - base standard deviation of the Gaussian receptive fields
- σ_*ratio*_ - the eccentricity scaling factor of the Gaussian receptive fields' standard deviation

While the generated tessellation does not exhibit obvious classical log-polar spirals, due to the stochastic nature of its production, In Figure 2, Left, we can observe that, at least subjectively, the annealed retinal tessellation appears similar to the spatial distribution of cone receptors in the human retina itself (Sawides et al., 2016). Regarding the locally random nature of the retina tessellation, has been reported that the stochastic, non-uniform placement of retina photoreceptors appears to contribute to the image sampling process by transforming aliasing artefacts to appear more similar to a noise component which in turn can be more readily accommodated in subsequent neural processing (Yellott, 1983).

**Figure 3:**
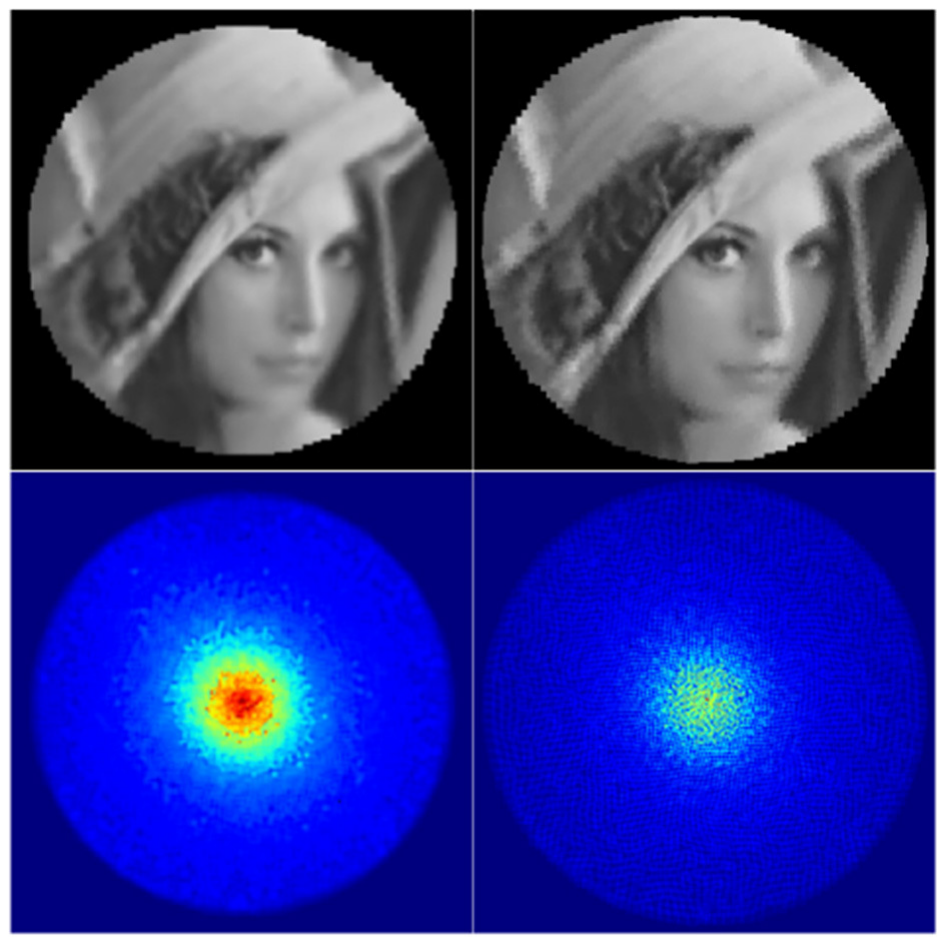
Backprojected retinal images and their retinas' Gaussian heatmaps. **Left:** a well-parametrised retina. **Right:** a badly parametrised retina.

The *dist*_5_ variable essentially defines the pixel distance between the two closest nodes in the tessellation by globally scaling the tessellation. The σ_*base*_ variable defines the base size and standard deviation of the Gaussian receptive fields: increasing it results in blurrier images, while overly decreasing it results in aliasing (jaggy) artefacts as visible in the top right image in Figure 3. The aliasing artefacts are especially apparent at the peripheries of the image. Finally, σ_*ratio*_ defines the difference between the receptive field sizes in the fovea and the peripheries of the retina.

The information captured by the retina can be visualised directly by generating a *backprojected retina image* (Figure 3, top row). It allows one to check visually whether the retinal subsampling appears sharp and free of aliasing artefacts. In order to obtain the backprojected image, every Gaussian receptive field kernel is projected onto an image-plane and scaled by its corresponding imagevector value. This image is then normalised by the *Gaussian heatmap image* (Figure 3, bottom row), which by itself is a useful visualisation of the spatial distribution of retina's receptive fields. The heatmap image can be obtained by simply projecting the receptive field Gaussians without scaling them by imagevector values. Aliasing artefacts in backprojected images can be produced by gaps between receptive fields in the retina's Gaussian heatmap image and can be suppressed by increasing the standard deviation of the projected Gaussian field. However, this comes at the expense of increasing the degree of blur the reconstructed image.

The receptive field parameters used were chosen manually by visually examining the Gaussian heatmaps and backprojected images for various parameter combinations. The objective being to produce heatmaps which are both free from “holes” mentioned above and also not significantly blurred. The chosen receptive field parameters are: *dist*_5_ = 1.0, σ_*base*_ = 0.4 and σ_*ratio*_ = 0.26 to produce a retina of 168 × 168 px in size.

## 3. Exploiting the Retino-Cortical Mapping Within Deep Learning

As described above, the basic retina samples an input image and produces an image vector as output, in a manner somewhat analogous to the optic nerve. However, this form of output is not compatible with current DCNN software environments as these have been designed to process regular image matrices as input. We have solved this compatibility issue by generating a *cortical image*. Even though our initial retina implementations did not fully exploit the potential data reduction efficiency of the retina image sampling approach, they did provide a straightforward and viable means to coupling the retina to any conventional DCNN architecture. The cortical image efficiency issue has now been substantially addressed in subsequent implementations (Shaikh, 2018) section 6.

### 3.1. Cortical Image Generation

#### 3.1.1. Requirements and Approach

We generate cortical images using Gaussian interpolated forward projection, a technique frequently employed in computer graphics. The receptive field centres used for retina sampling are first mapped into *cortical space* and then each imagevector intensity value is used to scale a small Gaussian kernel which is accumulated into the cortical image, centred on the appropriate cortical location. An alternative approach would be to map the back-projected retinal image pixel-by-pixel onto a new space; this however would be more computationally intensive and would not take advantage of the compression achieved by the imagevector. Additionally, the pixel-by-pixel approach would be much less flexible due to fewer parameters defining the behaviour of the process. It would also result in significant holes in the foveal region of resultant cortical images, similar to those reported in the work of Pamplona and Bernardino (2009). The approach taken eliminates the possibility of holes in the mapping as the size of the Gaussian kernel projected into the cortical image can be increased to provide the required degree of overlap and also suppress aliasing.

The cortical images should ideally preserve local angles, maintain a fairly uniform receptive field density and preserve all local information captured by the retina without introducing any noise. These requirements are laid out in order to enable the convolution kernels of CNNs to extract features from the resultant cortical image. The literature reviewed points toward a form of log-polar space as being the most appropriate approach since it has been shown to model with reasonable fidelity the mapping observed in the primate visual cortex (Schwartz, 1977). Mathematically, this mapping provides a plausible model, affording the key geometric features of observed cortical magnification of the fovea and compression of the peripheral visual field accordingly; it is also a conformal mapping, meaning that it preserves local angles.

#### 3.1.2. The Cortical Mapping

Retinal log-polar coordinates consist of θ, which is the angle about the origin (the centre-most point of the fovea), and ρ, which is the log of the distance from the origin. The *x* and *y* variables below represent retinal space Cartesian coordinates relative to the origin.

(1) $$\begin{array}{r} \rho =log\sqrt{x^{2}+y^{2}} \end{array}$$

(2) $$\begin{array}{r} \theta =atan2\left(y/x\right) \end{array}$$

**Figure 4:**
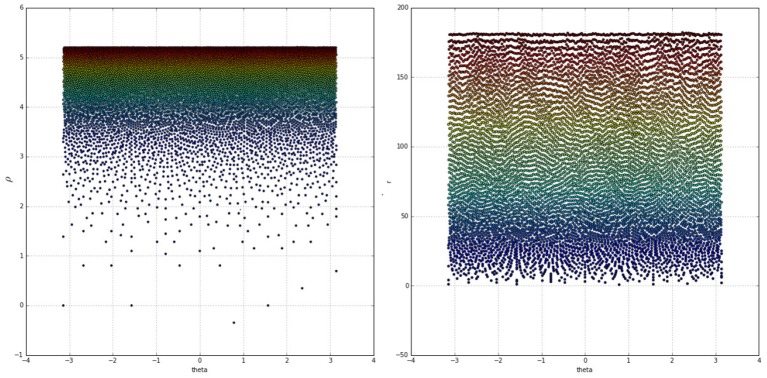
Colour coded receptive field centres mapped onto the log-polar **(Left)** and linear-polar **(Right)** spaces. Warmer colours indicate receptive fields closer to the peripheries, whereas colder colours indicate points closer to the fovea.

As evident in the left side of Figure 4 the log-polar space suffers from severe sparsity in the foveal region and excessive density at the peripheries. This has been mitigated by deviating from the approach proposed in the literature, removing the log operator from Equation (1) and switching to the “linear” polar space:

(3) $$\begin{array}{r} r=\sqrt{x^{2}+y^{2}} \end{array}$$

The right side of Figure 4 demonstrates the drastic improvement in node uniformity by switching to the polar space, although the foveal region is still undesirably sparse and the extreme peripheries are packed in tight rows. The uniformity of the polar mapping also suffers at *r* = 30 where the node density is too high compared to other regions. These issues have been resolved by adopting the approach from the work of Schwartz (1980) and adjusting the mapping with an α parameter while also splitting the retina tessellation vertically into two halves and mapping each half separately. This solves the singularity issue at the fovea and brings the mapping closer to the experimental data of activations in the visual cortices of different primates. The resultant coordinate equations for the cortical mappings are:

(4) $$\begin{array}{r} Y_{cort}=\sqrt{{\left(x+\alpha \right)}^{2}+y^{2}} \end{array}$$

(5) $$\begin{array}{r} X_{cort}=atan2\left(y/\left(x+\alpha \right)\right) \end{array}$$

**Figure 5:**
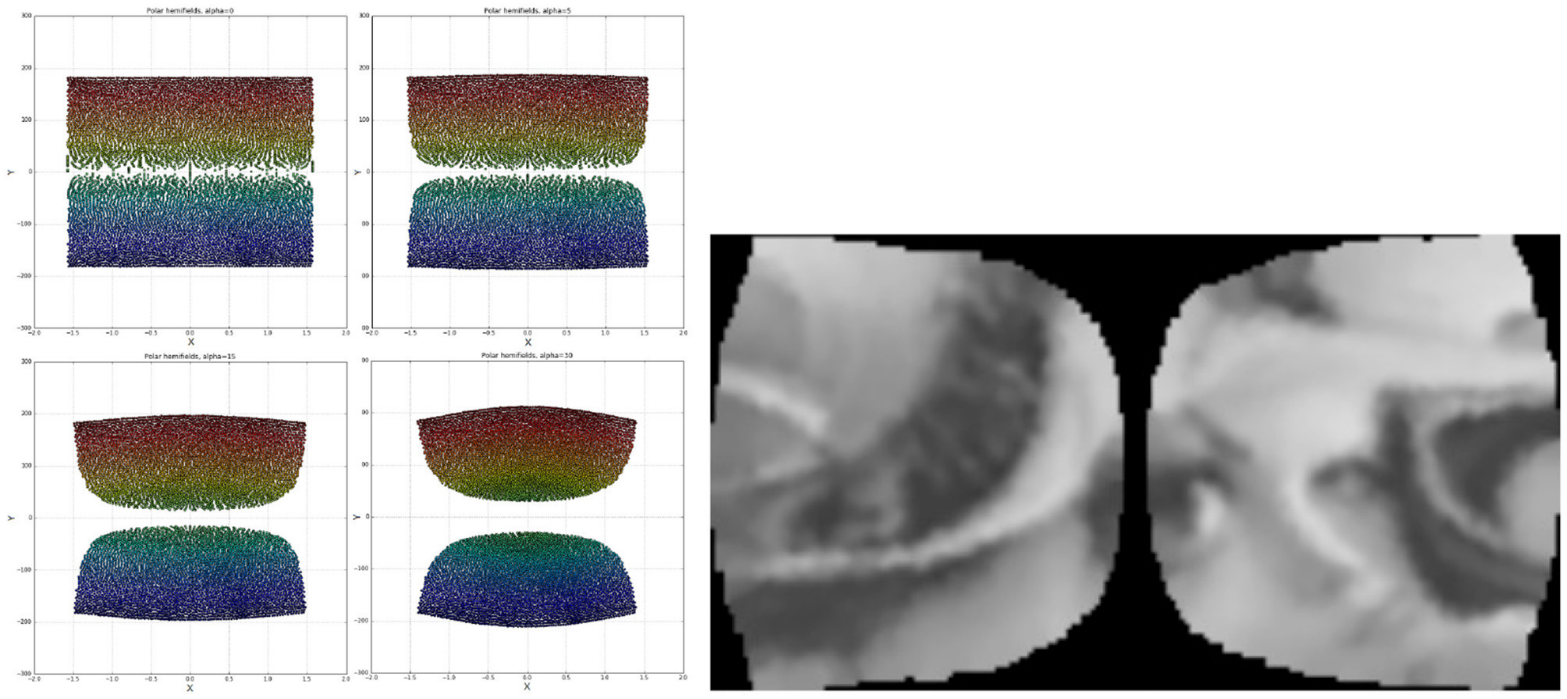
**Left:** Two hemifields of receptive field centres mapped onto a polar space. Going from top left to bottom right the α values are: 0, 5, 15, 30. The points are colour-coded based on the value of *sign*(*x*)^*^r. **Right:** A cortical image of the standard Lena image, equivalent to the optimal retinal backprojection from the left side of Figure 3.

As seen in Figure 5 the α parameter is added to the *x* coordinate to shift the tessellation's nodes away from the origin horizontally. In polar space this manifests itself by translating the nodes closer to *X* = 0, with the effect increasing logarithmically toward the foveal nodes at *Y* = 0. As the α parameter increases, the peripheral nodes (red and dark blue in Figure 5, Left) protrude proportionately; this is desirable as it addresses the issue of tightly packed rows of nodes from Figure 4. Accordingly, α sets the field of view of the quasi-linear region of the retino-cortical mapping. Note that in order for the left half of the retina to mirror the right one in Figure 5, Left its coordinates have been adjusted as follows:

(6) $$\begin{array}{r} X_{left}=-\sqrt{{\left(x-\alpha \right)}^{2}+y^{2}} \end{array}$$

(7) $$\begin{array}{r} Y_{left}=atan2\left(y/x-\alpha \right)-sign\left(atan2\left(y/x-\alpha \right)\right)*\pi \end{array}$$

It was decided that a value of α = 10 will be used as upon visual inspection it appeared the most uniform. Lower α values lead to an overly sparse foveal region, while higher values produced an overly dense region at *Y*≈±70, *X*≈0. In order to define the aspect ratio of cortical images the mean node distances along the x and y axes were equated.

Cortical images were produced by projecting Gaussians scaled by the associated imagevector value onto the appropriate nodes' locations with a sub-pixel accuracy of 1 decimal place. The resultant image was then normalised by pixel-wise division with the cortical Gaussian heatmap image, in a similar process to that used to generate retinal backprojected images in section 2.4. The cortical Gaussians were parameterised with σ = 1.2 and clipped at 7 pixels width. The two halves of the cortical image are also realigned to facilitate visual inspection.

The resultant cortical images, an example of which can be seen in Figure 5, Right, satisfy all the criteria for an acceptable input to a CNN: local angles are preserved, receptive fields are projected at a sufficiently uniform density and most of the local information captured by the retina is preserved without introducing any noise or artefacts. The cortical images have a resolution of 179 × 96 px, while a square that best fits the retina's resolution is 168 × 168 px large.

The cortical parameters were selected with a focus on reconstructing the foveal region of the image to ensure that the high-frequency content captured by the retina reaches the CNN. As a result the cortical images for this parameterisation display aliasing artefacts which are especially visible on images of synthetic objects with straight edges (Lena's hat in Figure 5, Right). Subsequent mappings, described in section 4, now address this issue.

While the visual data reduction achieved by this retina is approximately ×7, the compression ratio of the cortical transform is 1.64:1, due to the pixel interpolation required to generate a hole-free cortical image. However, our ongoing research has demonstrated that it is now possible to achieve the full data reduction potential for the approach section 6.

#### 3.1.3. Fovea Size and Hemifield Overlap

As we are inputting image files to the retina, as opposed to characterising a camera as input with specific FoV characteristics as determined by a lens etc., the concept of FoV in terms of visual angle does not apply to this retina as such, but in terms of *pixels*, 168px as described above. Specified as a fraction of the radial FOV, the retina parameterisation above has been chosen to generates a fovea of ~10%.

Due to the disjoint nature of the cortical mapping at the interface between the projected cortical hemifields, there is a loss of neighborhood information along the retina's vertical meridian, which is the ‘U' shaped border region in the cortical mapping as evident in Figure 5, Right. In future versions this could be resolved by having the two halves of the cortical mapping duplicate a set of nodes on the meridian, as found in the mammalian visual system which shares of the order of 1° of visual overlap along the vertical meridian in the retina, between each cortical hemifield in human vision. As both cortical hemifields are processed by a single DCNN in the implementation reported here, we believe that any information loss is likely to be minimal.

### 3.2. Gaze Control

**Figure 6:**
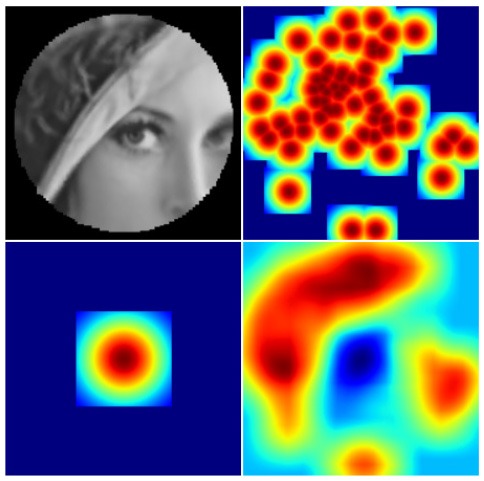
Going left-to-right, top-to-bottom: a retinal backprojection image; the equivalent region of the initial saliency map denoting SIFT feature locations; the equivalent region of the inhibition map resulting from the singular fixation; the final saliency map.

The implemented system follows Balasuriya (2006) by maintaining a saliency map of the input image, as well as an inhibition-of-return (IOR) map describing past fixations Figure 6. Initially the retina is fixated upon the centre of the image. To populate the saliency map the retinal backprojected image is scanned for SIFT features. A Gaussian is then projected at each feature's corresponding location in the saliency map, with the caveat that the projections do not sum with the saliency map's prior value, but override it if they are larger.

The location of each fixation is represented in the IOR map as an amplified Gaussian spanning the retina's foveal region. In order to determine the coordinates of the next fixation, the IOR map is subtracted from the saliency map, the result is blurred with a 37 × 37 averaging convolution kernel. The coordinates of the maximum value on the final saliency map determine the next fixation point. The result is satisfactory - the retina focuses on areas of the input image with the most corners present, and does not re-fixate upon the same location.

### 3.3. Validating the 4196 Node Retina

A dataset suitable for training and evaluating retina-integrated DCNNs (RI-CNNs) has to meet a set of requirements: the object of interest has to occupy most of the image in order to maximise the likelihood of the gaze control algorithm producing good fixations. Ideally, said object would be either segmented or cropped out from the background. The images have to be large enough to take advantage of the retina, but not too large so that a single fixation of the retina captures a reasonable proportion of the object of interest. In order to prevent the classification task from being trivial, each object class in the dataset should share a subset of its visual features with at least one other class, meaning that the classes should be somewhat similar to each other.

**Figure 7:**
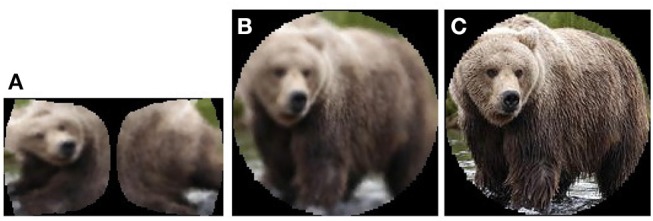
An example *Brown Bear* image from each of the three subsets **(A–C)**.

In order to fulfill the above requirements, a new dataset was created by selecting and pre-processing the appropriate classes from ImageNet (Deng et al., 2009). This dataset consists of three subsets: subset A is made up of cortical images (Figure 7, left), subset B is retinal backprojected images (Figure 7, centre) and subset C consists of the conventional images of each fixation, masked with the retinal lens (Figure 7, right).

The object categories selected for the classification task are *Basketball Hoop, Brown Bear, Keyboard* and *Racoon*. The similarities between *Brown Bear* and *Racoon* (furry animal), *Basketball Hoop* and *Keyboard* (synthetic object with a grid-like key feature) helped ensure that the classification task is not trivial. The class objects were cropped out from their original images using the bounding boxes provided in ImageNet. The resultant images passed automatic selection that ensured the images were not too small (*width, height* > 75, 75) or too long (1/3 < *width*/*height* < 3), and were then processed by appropriate parts of the retina pipeline to produce the three subsets. In order to correct a large imbalance between the class frequencies, the number of retina fixations was varied per class. After the subsets were produced, they were manually edited to remove exceptionally bad fixations and false positives resulting from incorrect labels assigned in ImageNet. The final image counts in the dataset can be seen in Table 1, Left.

**Table 1:**
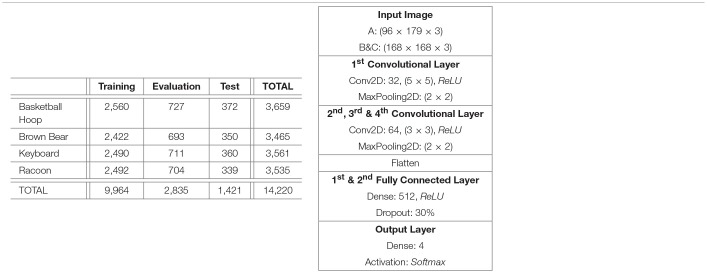
**Left:** Per class and per split fixation image counts. The numbers are consistent across all 3 subsets of the dataset. **Right:** DCNN architecture for 4,196 node retina.

### 3.4. 4196 Node Retina Results and Discussion

In order to evaluate the performance of the retinal subsampling mechanism and the cortical image representation in isolation, three DCNNs were trained, each with the same architecture but each using a different subset of the dataset built in the previous section. The DCNN architecture used, Table 1, Right, was chosen by trialing various architectures to maximise their performance over the cortical image dataset. The Keras 2.0.2. library was chosen as the Deep Learning platform used in this work. A relatively simple DCNN architecture was chosen in this pilot study, as our priority was to achieve benchmark classification performance testing, as opposed to optimisation of the recognition network.

We employed the Adam Kingma and Ba (2014) optimiser and a *categorical cross-entropy* loss function when training the DCNN. Improvements in validation accuracy were monitored and training was terminated automatically when this was no longer productive. L2 regularisation of strength λ = 0.02 was applied to the internal fully connected layers to prevent overfitting, however that value could most likely have been increased as the model continued to display signs of overfitting. The key figures from the training process are:

- **Network EVAL-A**, using (96 × 179) cortical images, reached its peak performance **(validation loss = 0.605, validation accuracy = 82.26%)** after **16 epochs**.
- **Network EVAL-B**, using (168 × 168) retinal backprojected images, reached its peak performance **(validation loss = 0.493, validation accuracy = 86.14%)** after **21 epochs** of training.
- **Network EVAL-C**, using (168 × 168) conventional images, reached its peak performance **(validation loss = 0.488, validation accuracy = 87.51%)** after **25 epochs** of training.

**Figure 8:**
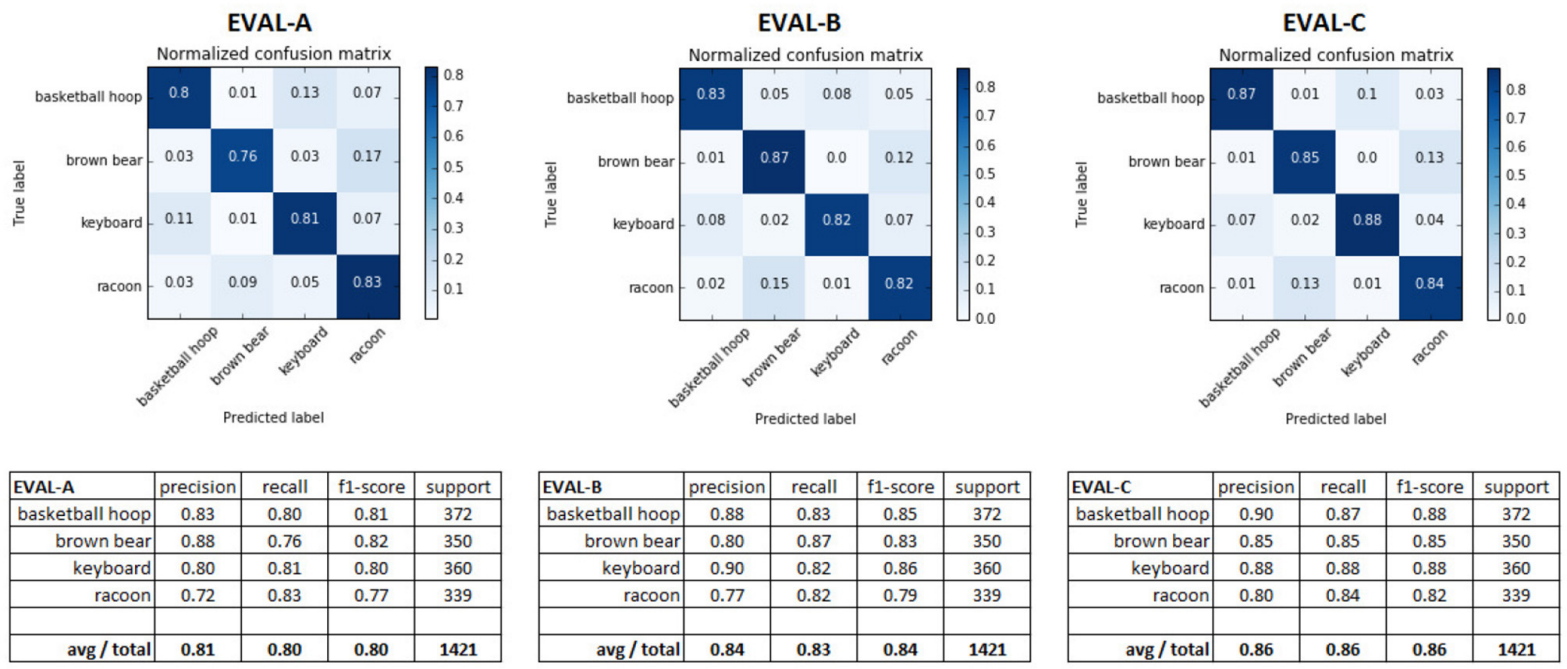
Confusion matrices and different performance metrics of the three DCNNs evaluated against the appropriate test sets.

The results from evaluating the networks against the test set (Figure 8) show that both applying the full retino-cortical transform and the retinal subsampling lead to a modest decrease in the DCNNs' performance. The network trained on conventional images performed the best, with an average F1 score of 0.86; the network trained on retinal images landed an F1 score of 0.84 while the cortical images network had an F1 score of 0.80 showing that remapping the image from the retinal to the cortical space was the most damaging aspect of the retino-cortical transform. As seen in the matrices in Figure 8, the majority of the networks' confusion is between the classes sharing similar key features.

Although the retina has reduced classification performance, the gap between the different networks' performances is not excessive and the network EVAL-A has successfully demonstrated the learning capacity of convolutional neural networks for images in the cortical view while achieving a 7-fold visual data reduction and a 1.64 DCNN input compression ratio, fulfilling the main objective of this pilot study.

When interpreting the above pilot results, it should be kept in mind that it is possible that manually editing the dataset may not have removed all false positive and bad fixation images. The dataset is also not optimally large, and the input images are often small resulting in a number of fixation images being mostly empty, i.e., lacking visual input by fixating on the black border surrounding the image region sampled by the retina.

## 4. A High Resolution 50,000 Node Real-Time Retina Implementation

In the light of the above experiment it was decided to develop a a high-resolution 50K node retina (~10% fovea size by radius) to achieve a visual data-rate reduction of approximately ×16.7. While initial implementations achieved compression ratios of the order of ×3, even this level of data reduction allows current DCNN implementations to process images approaching megapixel resolution *in a single pass*, described in section 5. A compression ratio of ×11.5 has now been achieved for this retina, section 6. Accordingly, the need to apply a DCNN window that is scanned over an image pyramid is no longer required and this greatly improves the overall efficiency of the DCNN Ozimek and Siebert (2017), Ozimek et al. (2017), and Hristozova et al. (2018). This retina implementation has been optimised in terms of reducing aliasing artefacts resulting from retinal sampling and cortical image production. Examples of cortical and back-projected retina images are shown in **Figure 10**.

Given the level of computation required to execute the new 50K node retina and the need to support subsequent retina developments and real-time applications based on retina sampling, we decided to implement a hardware accelerated retina. This implementation has been written in CUDA C to execute on the NVIDIA series of Graphics Processor Units (GPUs) and is capable of sampling an RGB image to produce a triple of RGB image vectors in 39.1 ms, executing on an NVIDIA GTX 1080 Ti GPU (Balog, 2017).

## 5. Human Eye-Tracking Based Egocentric Perception

**Figure 9:**
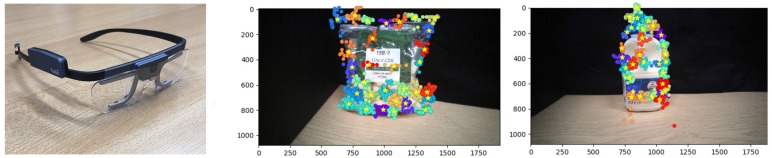
**Left:** Tobii Pro2 Eye Tracker Glasses; **Right:** Fixation clustering following homography alignment.

We have undertaken an experiment (Hristozova, 2018), to combine the high-resolution 50K node software retina (Balasuriya, 2006; Ozimek and Siebert, 2017; Ozimek et al., 2017) with a custom designed DCNN architecture [based on DeepFix (Kruthiventi et al., 2017)] coupled to an image stream collected by Tobii Pro 2 eye-tracking glasses (AB, 2017) (Figure 9) worn by a human observer. Our objective is to demonstrate that we can achieve state-of-the-art recognition performance using our high-resolution retina implementation while also achieving efficiency gains. In addition, we wanted to investigate the potential to adopt a human observer for directing our software retina's gaze to thereby construct a truly egocentric perception system suitable for both humans and robots. Our final objective for this experiment is to demonstrate that it is possible for a human operator to collect appropriate training data for a software retina-based egocentric perception (Siebert et al., 2016; Ozimek et al., 2017) system simply by looking at objects. These objects may then be recognised in images collected by a human observer using eye tracking glasses, or a machine observer equipped with a saliency model to direct visual gaze.

### 5.1. Eye Tracking Pipeline

Our processing pipeline comprises four stages: image capture, fixation cluster extraction, retina transformation and DCNN processing. Following image collection using the Tobii glasses, described below, the images for the observations of each object are composited, in order to allow the individual fixations associated with each observation (fixation) to be overlaid on a single reference image. This approximate alignment was initially achieved by means of SIFT descriptor matching and extraction of the inter-image homographies, however, we discovered that simple head stabilisation was sufficient to achieve the required image registration. *K*-means clustering is then applied to these co-referenced fixation locations, where *K* has been set to 1% of the number of fixations in the observations for the current object class. This both reduces the number of fixated training images to manageable numbers and also selects locally coherent clusters of fixations, whose convex hulls are used to locate the software retina within the input image, as shown in Figure 9. The smaller cortical images produced by the retina are then input to the DCNN for both training and inference purposes.

### 5.2. Eye Tracking Data Collection

A custom interface was developed to allow an operator to control the acquisition of images using the Tobii Pro 2 eye tracking glasses. The two observers who participated in this experiment were instructed to look at locations on the surface of each object which seem particularly salient, or diagnostic of each object's identity, when collecting images. As mentioned above, it was necessary to stabilise observer's head by resting their chin on a desk surface while observing each object using the Tobii glasses and also by their consciously minimising any head movement. A data set of over 26,000 images was collected using the Tobii glasses, split into three categories: Training, Validation and Test. Each of these categories contains 9 object classes: Eggs, Gnocchi, Juice, Ling, Milk, Rice, Strep, VitC and Yogurt. Each of the data categories comprises the following proportion of the total data: Training 80%, Validation 18% and Test 2%.

### 5.3. Building a DCNN for Classifying Eye Tracking Image Data

Inspired by Kruthiventi's DeepFix Kruthiventi et al. (2017) network we developed a hand-optimised DCNN architecture comprising seven *convolutional layers(CLs)*, as summarised in Table 2. The first two CLs have been configured with 32 convolution filters, the second two CLs with 64 convolution filters, the fifth CL comprises 128 filters while the final two CLs comprise 256 filters. In all seven convolution layers the filters are 3 × 3 in size and each of these layers is coupled by a 2 × 2 ReLU max pooling function. Thereafter, the output of the last pooling layer has been flattened prior to being coupled to three *fully connected (FC)* layers, each comprising 132 nodes and a final fully connected layer configured with nodes corresponding to the number of output classes, in this case 9. Each FC layer had a rectifier activation function. While the activation function of the output layer was set to *softmax*, to provide classification values ranging from 0 to 1. The classifier was optimised using *stochastic gradient descent* and *categorical cross entropy* was used to compute the loss function. Drop-out was set to 50% and applied only once, after the first fully connected layer.

**Table 2:**
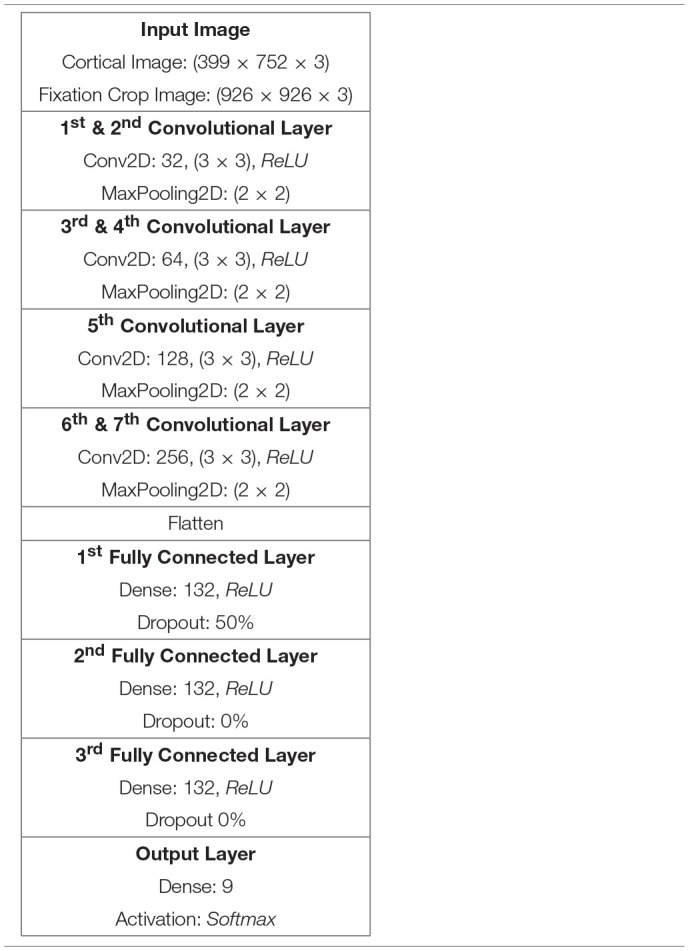
DCNN architecture for Cortical and Fixation Crop image classification using the 50K node retina.

### 5.4. Cortical and Fixation Crop Image DCNN Validation

**Figure 10:**
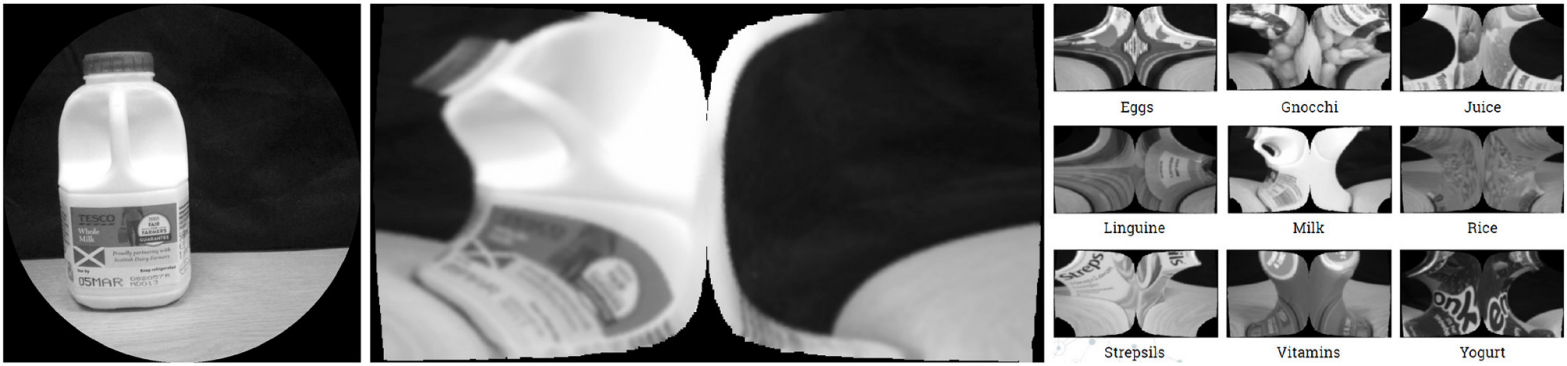
**Left-Right:** Fixation Crop Image, Corresponding Cortical Image (up-scaled), Example Cortical Images of the 9 Object Classes Captured.

In order to compare the performance obtained when pre-processing images using the software retina, as opposed to classifying standard images, two DCNN models were trained: the first with fixation crop images of size 926 × 926 px and the second with the cortical images of size 399 × 752 px. Examples of fixation crop and cortical images are given Figure 10. These images were also normalised prior to being input to the DCNN.

#### 5.4.1. Cortical Image DCNN Validation

**Figure 11:**
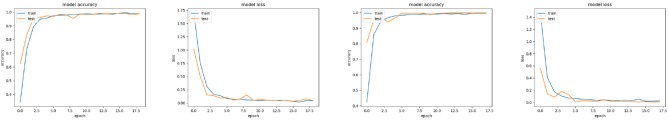
**Left-Right:** Cortical image accuracy, Cortical image loss, Fixation Crop image accuracy, Fixation Crop image loss, each versus training epoch.

The cortical image classification DCNN model was trained using 270 steps per epoch (total number of images/batch size), where the batch size was set to 64 and the training and validation data sets comprised 21,310 and 4,800 images respectively. This model required 55 min processing time to execute 18 epochs and produced 98% validation accuracy. Figure 11 shows the accuracy and loss respectively. 6 s of processing time were required for this model to classify the data in its test set, resulting in an average accuracy of 98.2%.

#### 5.4.2. Fixation Crop Image DCNN Validation

As illustrated in Figure 10, the fixation crop of an original image contains the retina's field of view, but retains the full image resolution. In order to benchmark the performance of the cortical image DCNN classifier, a DCNN model was trained using the full-resolution fixation crop images. In this case 1217 steps per epoch were used (again total number of images/ batch size) where the batch size is set to 16 and the training and validation data sets comprised 19,485 and 4,390 images respectively. The batch size had to be reduced to 16 from 64 used for the cortical image DCNN, because the increased numbers of pixels in the fixation crop images invoked a TensorFlow memory exhaustion error at any larger batch size. The resulting accuracy and loss are shown on Figure 11. This model required 2 h and 30 min to execute 18 epochs and produced 99% validation accuracy. 12 s of processing time were required for this model to classify the data in its test set, resulting in an average accuracy of 99.5%.

### 5.5. Eye Tracking Based Retina Validation Discussion

From the above results, use of retina pre-processing has reduced the training time for the DCNN from 150 min (using full-resolution input images) to 55 min using cortical input images. Since the full-resolution images are ×3 larger than the cortical images, the training batch size had to be reduced to 16 images for training with full-resolution images, as compared to a batch size of 64 images when training with cortical images. This improvement in data efficiency came at the expense of an average classification performance reduction of 1.3%. Even this modest reduction in performance has now been removed in subsequent work that uses a more efficient cortical image generation process, Section 6.

In both the cortical image and fixation crop classification experiments, the validation accuracy obtained for each classifier is close to the corresponding training accuracy result. Given the limited range of observations when the observer's head is constrained to be stationary, the captured images will be correspondingly similar. However, due to the non-linear nature of the retina transformation, the cortical images continue to exhibit significant variation in appearance when undertaking small saccades when exploring an object. Since small translations in retina space result in rotations in the cortex, this appears to afford a degree of *implicit data augmentation* when training the DCNN in cortical space, i.e., the rotated versions of patterns in the cortex provide a wider range of training data in terms of pattern-space than the corresponding retinal observations would otherwise.

## 6. Retina Developments

Our primary technological objective is to realise fully the potential gains of the combined retina-DCNN approach, its integration within mainstream robot visual processing DCNNs and underpin practical low-cost visual sensors for autonomous systems. However, any new image format must be adequately supported to be of any practical utility. Accordingly, the following section outlines recent work to advance the development of the retina and to support applications based on the retina image format.

### 6.1. Retina Filter Models

In order to further investigate the utility of mimicking the human visual system we extended the software retina to incorporate a functional model of single and double-opponent retina cells that has potential to improve data efficiency through sparsification, partial figure-ground separation, texture description and contour isolation Figure 13.

Retina cell models for computer vision purposes typically comprise basic implementations of the Marr-Hildreth difference of Gaussians operator (Marr and Hildreth, 1980), perhaps incorporating a filter-bank operating at a number of different spatial scales. An example of a more complex retina model is reported by Gobron et al. (2007) who implement the coarse functional properties of the retina using cellular automata and accelerate their model using GPU programming in the OpenGL environment. The general function of the 5 different retinal neuron types has been expressed, while other architectural features of the retina, such as the foveated topography of its neurons and their receptive field response profiles, are not represented in this model. The output from this retina is a depth-like contrast image that is sensitive to motion. While this model might prove valuable in a retina inspired edge detection task, it is incompatible with the space-variant retina architecture adopted here.

Given the limitations of the above retina models based on uniform sampling, we have developed our own model that incorporates a basic functional implementation of single and double opponent retinal ganglion cells which vary in their receptive field size as a function of eccentricity. This model utilises a high resolution (50K node), GPU accelerated retina and is based on the work of Gao et al. (2013). The single and double colour opponent ganglion cells compute a colour space potentially capable of simplifying texture perception, colour constancy and ground-figure segmentation owing to the combination of both colour and texture features in a single mapping (Saarela and Landy, 2012).

Our model implements single and double opponent receptive fields using difference-of-Gaussians kernels, with the surround receptive field Gaussian having the *sigma* parameter three timer larger than the centre receptive field Gaussian, based on physiological findings regarding the structure of cat retinal ganglion cells (Rodieck, 1965). The model supplements the RGB colour space with a yellow channel (*(r + g) / 2*) in order to simulate four Type-2 single opponent cell species: centre opponent *r-g* and *b-y*, and surround opponent *g-r* and *y-b*.

A single opponent cell is simulated by applying retinal sampling twice: once using the standard retina (to sample the centre fields) and then a second time using a retina with Gaussian receptive fields with a *sigma* scaled by a factor of three (to sample the surround fields). Single opponent cells of any species can then be implemented efficiently by simply subtracting the appropriate centre and surround image vector responses. The differential image vector responses simulating single opponent cells are then added together in a spatially opponent manner in order to produce colour channels that model the double opponent cells.

In order to remain faithful to cortical physiology the summation of the type 2 single opponent cells is *leaky*, meaning that the centre receptive field is not perfectly balanced with the surround receptive field (Shapley and Hawken, 2011). To realise this the surround receptive field has been scaled by *k = 0.9* (Gao et al., 2013). Such leaky fields imply that absolute intensity or colour stimuli will generate a response and therefore be encoded along with the differential response of the antagonistic fields.

**Figure 12:**
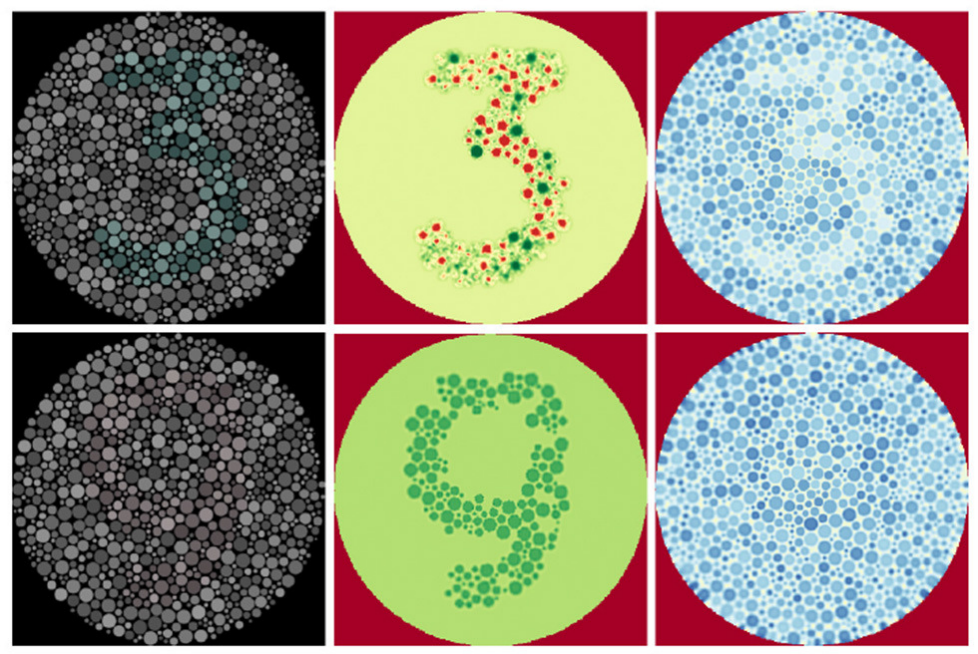
Low contrast colour-blindness tests processed using our double-opponency model. Reproduced by permission of EnChroma Inc., from enchroma.com. **Left:** Original images. **Centre:** Retinal backprojections of the *red-green* double opponent cells, coloured using a divergent colour map. Red indicates negative values, yellow indicates values near zero, and green stands for positive values. **Right:** retinal backprojections of the *blue-yellow* double opponent cells, coloured using a divergent colour map. Red indicates negative values, yellow indicates values near zero, and blue stands for positive values.

For visualisation purposes the outputs of the double opponent cells were backprojected (section 2.4) onto the image plane using the centre receptive fields. Figure 12 shows the resultant images and demonstrates the improved figure-ground differentiation using colour blindness tests. Since negative-valued signals are processed in the brain using separate pathways to positive-valued signals, the outputs of the double opponent cells should be split into their negative and positive components using a threshold before further processing. (To enhance visualisation of the filter outputs, this step was omitted when generating the images in Figure 12 and diverging colour maps were employed instead).

**Figure 13:**
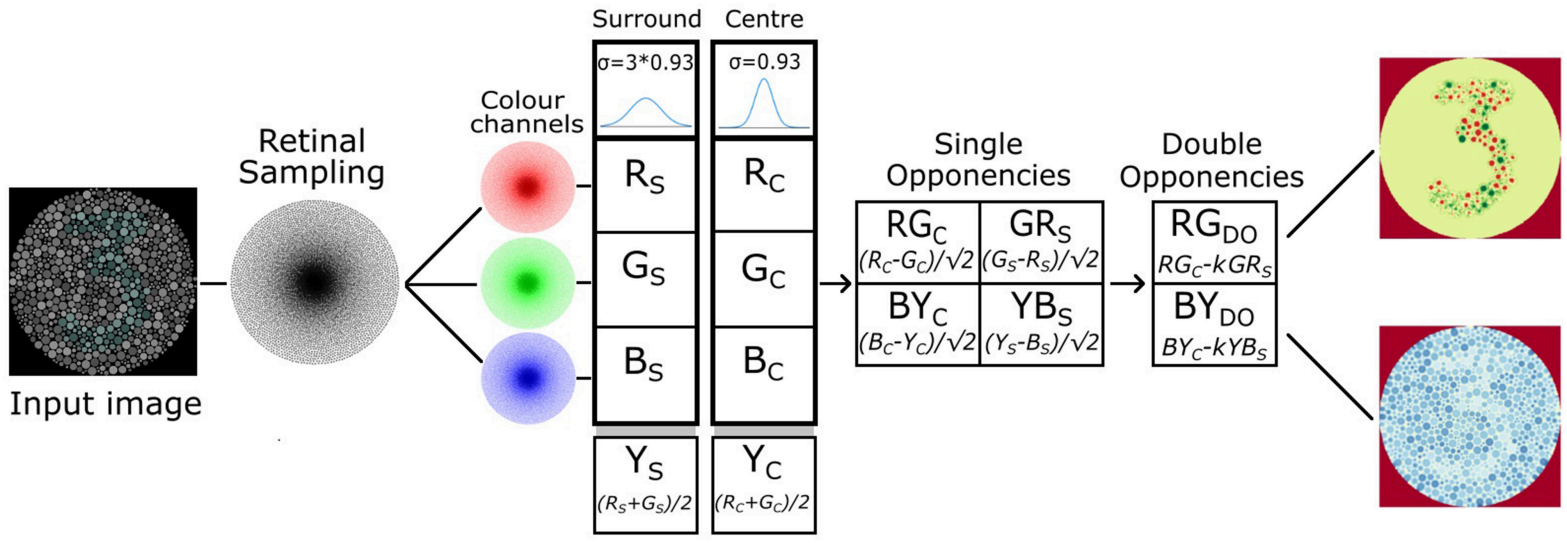
Single and double opponent retina cell model implemented for our 50K node retina, based on the work of Gao et al. (2013).

### 6.2. Real-Time Tracking Retina

The GPU accelerated implementation of the 50K node retina has been used to demonstrate real-time gaze control in a target tracking task (Boyd, 2018). In this experiment we trained a retina pre-processed DCNN pipeline with example tracking data based on a centroid colour tracker following an orange coloured target set against a dark background. The DCNN system learned to drive the Baxter Research Robot's wrist camera using pan-tilt signals learned from the cortical images input to a custom DCNN we designed. Accordingly our DCNN was able to regress, directly in real time, from cortical space, the appropriate pan-tilt activation to allow the robot's wrist camera to track the coloured target.

### 6.3. Retina Efficiency

We have made an initial attempt to tackle the fundamental issue of how best to couple the retina directly to the DCNN to obtain the maximum data efficiency. Shaikh (2018) discovered that simply subsampling the cortical image a network input data reduction of ×11.5 can be achieved, without compromising performance when undertaking the eye tracking based classification task described in section 5. Furthermore, by adopting a scattered datapoint gridding algorithm, developed for astronomy data processing purposes (Winkel et al., 2016), he was able to produce cortical images that yield both a network input data reduction of ×10.8, and also a modest increase in classification accuracy.

Generating an optimal cortical image offers two strong advantages: Firstly, the retina pre-processor remains fully compatible with existing DCNN processing architectures developed for visual processing. Secondly, by retaining the continuity of retinotopic map explicitly within a cortical image, it simplifies the task of interpreting and devising and debugging new “retinised” visual processing DL networks and allows efficient shared convolutions to be applied to the CNN layers which process the input cortical image.

### 6.4. Retina Sensors for Robotics and Egocentric Perception

In order to develop self-contained smartphone-based retina sensors for robotics and egocentric perception applications and a convenient method for capturing training data by non-expert users, we have implemented the 4196 node retina on an Apple iPhone (Wong, 2017). This comparatively small retina samples a patch in an image captured by the iPhone's camera and SIFT, descriptors are extracted from the cortical image to direct the next retinal fixation location of the next in conjunction with a simple inhibition of return algorithm. The concept has been extended to port the 50K node retina to both iPhone (Vinickis, 2018), and Android (Yang, 2018), smartphone platforms. In the Android implementation no gaze-control has been implemented, instead relying on the user to direct the cameras to record compact image vectors, which can then be stored on the cloud.

A fundamental characteristic of any vision system based on a space variant retina architecture is that it must be directed appropriately to sample the scene. Commercial high-resolution pan-tilt security cameras have the potential to serve as low-cost imaging sensors that can be steered under computer control. We have constructed a software interface to a standard networked pan-tilt security camera controller (Zhou, 2018), to allow this to serve within an active autonomous vision system using retina processing and cortex-based gaze control algorithms.

Finally, to manage the data generated by retina-supported camera systems, we have developed a prototype software tool to allow editing and formatting prior to training DCNN systems (Fulton, 2018).

## 7. Conclusions and Ongoing Work

We have confirmed the utility of the functional architecture of the human visual pathway, as predicted by Schwartz and others, by investigating retino-cortical mapping models within implementations of computer vision systems based on Deep Learning. Our primary experiment has shown that it is possible to make substantial data efficiency gains in terms of training computation, DCNN sizes, and inference rates by pre-processing images using our biologically inspired retina-cortex mapping that affords both visual data reduction and also a degree of scale and rotation invariance. It should be noted that we have not yet measured directly any scale and rotation invariance afforded by our retino-cortical mappings in the DCNNs we have trained to date, but plan to in future investigations.

While our initial attempt at demonstrating the concept, based on a 4,196 node retina, achieved only modest data reduction gains, our 50K node retina is now able to achieve a ~ × 16.7 visual data reduction and network input reduction of ~ × 11.5, while maintaining state-of-the-art performance in a classification task. We also demonstrated the viability of using human fixations to provide gaze-control for this 50K node software retina, which generated cortical images that were processed by means of our own DCNN model to obtain excellent classification performance on a database of 9 object classes. This approach has also demonstrated substantial reductions in DCNN training times and critically has provided the means for a DCNN to process an image of 930 × 930 px image for training or prediction *in a single pass*, while executing on a standard consumer-grade GPU.

Given that the initial experiments we report here have utilised the most basic of DCNN architectures, our current research is focussed on building improved DCNN models to process the cortical images generated by the retina. Inspired by predictive encoding brain theory, deep predictive coding networks Wen et al. (2018) which adopt feedforward, feedback and recurrent connections and have been reported to consistently outperform feedforward only DCNNs when undertaking object recognition and would therefore appear to be a promising architecture to investigate coupling to the retina.

The combined retina-DCNN approach is currently being investigated in a number of contexts for visual processing required by robotics systems for tracking and grasping and in the perception of egocentric imagery. Accordingly, our laboratory is developing the necessary infrastructure in terms of camera interfaces and data management tools to support DL-based visual processing based on the software retina. Furthermore, the software retina mapping also has potential for use in visual models developed to interpret human fMRI imagery for visual cortex modelling purposes.

Given both the visual data reduction potential of the retino-cortical mapping and the consequent implications for low-cost robotics and egocentric visual processing systems, especially mobile systems using smartphone or embedded processing hardware, the authors believe that this approach will underpin both a new wave of biologically motivated computer vision research and make possible vision-based products requiring high-resolution imaging that would otherwise be impractical to achieve using currently available hardware technology.

In conclusion, researchers have been attempting to harness log-polar visual mappings for over four decades and the authors believe that work we have presented is the first viable demonstration of using the retino-cortical mapping within a general purpose visual processing methodology.

## Data Availability

The datasets analysed for this study can be found in doi: 10.5525/gla.researchdata.744.

## Author Contributions

PO implemented the retina codes, contributed associated text and undertook the 4K retina study. NH undertook the Eye tracking study, contributed associated text. LB implemented the GPU accelerated retina. JS proposed the retina-DCNN approach, lead preparation of manuscript, supervised the research presented.

### Conflict of Interest Statement

The authors declare that the research was conducted in the absence of any commercial or financial relationships that could be construed as a potential conflict of interest.
